# Identifying the exposure of taxonomic, functional, and phylogenetic diversity of steppe birds to renewable energy development

**DOI:** 10.1111/cobi.70291

**Published:** 2026-04-19

**Authors:** Pablo Medrano‐Vizcaíno, François Mougeot, Beatriz Arroyo, Gerard Bota, David Giralt, Laura Maeso‐Pueyo, Carlos A. Martín, Manuel B. Morales, Pedro P. Olea, Juan Traba, Ana Benítez‐López

**Affiliations:** ^1^ Instituto de Investigación en Recursos Cinegéticos IREC (CSIC‐UCLM‐JCCM) Ciudad Real Spain; ^2^ Department of Computer Science Oklahoma State University Stillwater Oklahoma USA; ^3^ Biodiversity Management and Conservation Program Forest Science and Technology Centre of Catalonia (CTFC) Solsona Spain; ^4^ Department of Biogeography and Global Change MNCN‐CSIC Madrid Spain; ^5^ Department of Biodiversity, Ecology and Evolution, Faculty of Biological Sciences Universidad Complutense de Madrid Madrid Spain; ^6^ Department of Ecology Universidad Autónoma de Madrid Madrid Spain; ^7^ Centro de Investigación en Biodiversidad y Cambio Global (CIBC‐UAM) Universidad Autónoma de Madrid Madrid Spain

**Keywords:** biodiversity dimensions, functional diversity, photovoltaic, phylogenetic diversity, strategic planning, utility‐scale solar energy, dimensiones de la biodiversidad, diversidad filogenética, diversidad funcional, energía solar a gran escala, fotovoltaico, planeación estratégica

## Abstract

Biodiversity is globally threatened by human impacts, including land‐use transformation and climate change, which has prompted a rapid transition from fossil fuels to cleaner energy sources, such as photovoltaic (PV) energy. However, utility‐scale PV plants require vast areas and can lead to conflicts with biodiversity conservation, making strategic planning essential. We used steppe birds, a highly threatened group occurring in lands potentially suitable for PV plants, as a model to evaluate the spatial overlap between conservation priorities and PV infrastructure. We quantified and mapped taxonomic (TD), functional (FD), and phylogenetic (PD) diversity of 26 species across one of the main strongholds of European steppe birds (mainland Spain and Balearic Islands) to identify prioritization scenarios that best preserved all steppe bird diversity facets (TD, FD, and PD) and steppe birds of conservation concern. We generated a multifaceted diversity map based on the combination of TD, FD, and PD and overlaid existing PV infrastructure to determine bird exposure areas (i.e., high multifaceted diversity with current high PV occupancy) and PV avoidance areas (high multifaceted diversity with low or no current PV occupancy). The prioritization scenario that combined TD, FD, and PD retained 68.5% of TD, 75.5% of FD, and 59.7% of PD and 62.8% of areas with species of conservation concern, providing a more balanced representation across biodiversity facets than other prioritization scenarios. PV infrastructure occurred in 53.1% of high‐multifaceted‐diversity cells. Exposure areas (7.2% of the study area) were mainly concentrated in central, southern, and northwestern Spain, and PV avoidance areas (19.9% of the study area) were concentrated in northern, central, and central‐western Spain. Our method is adaptable to other species, communities, and regions and offers a robust framework for balancing development projects with biodiversity conservation.

## Abstract (es)

Identificación de la exposición al desarrollo de energías renovables de la diversidad taxonómica, funcional y filogenética de las aves esteparias

**Resumen:**

La biodiversidad está amenazada a nivel mundial por el impacto humano, que incluye la transformación del uso del suelo y el cambio climático. Esto ha impulsado una rápida transición de los combustibles fósiles a fuentes de energía más limpias, como la energía fotovoltaica (FV). Sin embargo, las plantas fotovoltaicas a gran escala requieren extensas superficies y pueden generar conflictos con la conservación de la biodiversidad, por lo que resulta esencial una planificación estratégica. Utilizamos las aves esteparias, un grupo altamente amenazado que habita en terrenos potencialmente aptos para plantas fotovoltaicas, como modelo para evaluar la superposición espacial entre las prioridades de conservación y la infraestructura fotovoltaica. Cuantificamos y cartografiamos la diversidad taxonómica (DT), funcional (DF) y filogenética (DFi) de 26 especies en uno de los principales bastiones de las aves de la estepa europea (España peninsular y las Islas Baleares) para identificar escenarios de priorización que preservaran mejor todas las facetas de la diversidad de las aves de la estepa (DT, DF y DFi) y las aves de la estepa de interés para la conservación. Generamos un mapa de diversidad multifacética basado en la combinación de las tres diversidades y lo sobrepusimos a la infraestructura fotovoltaica existente para determinar las zonas de exposición de las aves (es decir, alta diversidad multifacética con una elevada ocupación fotovoltaica actual) y las zonas de evitación (alta diversidad multifacética con una ocupación fotovoltaica actual baja o nula). El escenario de priorización que combinaba DT, DF y DFi conservaba el 68.5 % de DT, el 75.5 % de DF y el 59.7 % de DFi, así como el 62.8 % de las áreas con especies de interés para la conservación, lo que proporcionaba una representación más equilibrada entre las facetas de la biodiversidad que otros escenarios de priorización. La infraestructura fotovoltaica se encontraba en el 53.1 % de las celdas de alta diversidad multifacética. Las áreas de exposición (7.2 % del área de estudio) se concentraron principalmente en el centro, el sur y el noroeste de España, y las áreas de evitación (19.9 % del área de estudio) se concentraron en el norte, el centro y el centro‐oeste de España. Nuestro método es adaptable a otras especies, comunidades y regiones, y ofrece un marco sólido para equilibrar los proyectos de desarrollo con la conservación de la biodiversidad.

## INTRODUCTION

Human activities alter biodiversity, with the most prominent threats being habitat loss caused by land transformation and climate change (IPBES, 2019; Powers & Jetz, 2019). In response, global efforts to reduce carbon emissions have accelerated the transition from fossil fuels to cleaner energy sources, such as solar and wind power, which produce lower or no direct greenhouse gas emissions (Chien et al., 2022). Photovoltaic (PV) solar energy accounted for nearly one third of the total installed renewable energy capacity in the world in 2022, with projections of a 10‐fold increase by 2040 (Kruitwagen et al., 2021; Pourasl et al., 2023). At the regional level, the European Green Deal aims to achieve carbon neutrality by 2050 (European Commission, 2019) and to double PV capacity by 2030 (European Commission, 2022).

Although renewable energy projects aim to mitigate climate change, solar energy facilities require large land areas, which can lead to conflicts with other land uses, especially food production and biodiversity conservation (Dunnett et al., 2022; Hermoso et al., 2023; Serrano et al., 2020). In some regions, such as Europe, India, Japan, and South Korea, PV installations are expected to occupy between 0.5% and 5.2% of land by 2050 (van de Ven et al., 2021). This can result in wildlife habitat loss and additional negative impacts including soil erosion, pollution, altered microclimates, changes in light and hydrological regimes, and increased animal mortality from various sources, including collisions with PV panels (Dhar et al., 2020; Gómez‐Catasús et al., 2024; Lambert et al., 2022). Associated infrastructure, such as roads and power lines, can fragment landscapes and cause mortality (Guil & Pérez‐García, 2022; Medrano‐Vizcaíno et al., 2023). Indeed, abundance, species richness, and density of various taxa have been found to be lower in areas with PV installations compared to adjacent, undisturbed areas (Jeal et al., 2019; Montag et al., 2016; Visser et al., 2019).

In turn, some species may benefit from solar energy facilities when wildlife needs are considered (e.g., plants in hydrologically deprived environments, Liu et al., 2019; birds in arable farmlands, Copping et al., 2025). Nonetheless, the scientific evidence on PV impacts on biodiversity, whether negative or positive, is still sparse, focused on local‐scale (e.g., landscape and microhabitat) impacts, and geographically and taxonomically biased toward the United States and the United Kingdom, and arthropods and plant species (Gómez‐Catasús et al., 2024; Lafitte et al., 2023). Studies on vertebrate species with large‐scale ecological requirements and studies linked to habitats with topographic and climatic features targeted by PV developers are missing.

Assessing the impacts of renewable energy projects on biodiversity is crucial to ensure that sustainability and conservation goals are met (Gómez‐Catasús et al., 2024). A first step to avoid negative PV impacts is the implementation of proactive land‐use planning at regional or national scales based on ecological attributes for providing recommendations for spatial siting of renewable energy projects in areas that do not conflict with biodiversity (Hermoso et al., 2023). A wealth of research has focused on how PV systems intersect with important conservation areas (ICAs), such as protected areas, key biodiversity areas, and wilderness areas (Dunnett et al., 2022; Niebuhr & Sant, 2022; Santangeli et al., 2016; Sonter et al., 2020). Yet, these assessments fail to recognize that impacts on species and functional groups are uneven (Pérez‐García et al., 2022). For instance, highly mobile species, such as birds, can spend long periods outside ICAs (Boakes et al., 2019) and die from collisions with infrastructures. In the early stages of large‐scale solar energy development, approximately 9.9 birds/MW were estimated to die each year in the United States due to collisions with solar facilities (Walston et al., 2016). Because grasslands and agricultural lands tend to be less protected than forests, species linked to these habitats spend much of their life cycle outside ICAs; hence, they are disproportionately exposed to PV developments that may not have to comply with regulations linked to ICAs (Serrano et al., 2020). This problem deserves particular attention because even some steppe bird species (e.g., *Pterocles orientalis*) have been reported to become locally extinct due to poor PV plant planning (Bolonio et al., 2024).

Steppe birds are declining worldwide and are the most threatened group of birds in Europe (Burfield et al., 2023). These birds may be particularly vulnerable to PV developments as they inhabit low‐intensity flat agricultural areas, where many PV projects are currently being developed (Serrano et al., 2020). This scenario is exacerbated in Spain, a territory that contains a large proportion of the European steppe bird populations and that constitutes one of their main strongholds (Burfield et al., 2023; Medrano‐Vizcaíno et al., 2025). There, severe declines have been observed in recent years (Traba & Morales, 2019; Traba & Pérez‐Granados, 2022), reflected in notable changes in the threat status at the country and continental level for several species (Gómez‐Catasús et al., 2025; Medrano‐Vizcaíno et al., 2025). Indeed, the comparison of the Spanish Red Lists (SRL) of 2004 and 2021 (Madroño et al., 2004; SEO BirdLife, 2021) shows that 13 (50%) out of 26 steppe bird species have been upgraded to a higher conservation status. The threat status of steppe bird species could be further worsened by the Spanish National Integrated Energy and Climate Plan (PNIEC) for 2023–2030 (MITECO, 2024a), which plans an increase of approximately 65,000 MW of solar PV energy production by 2030 in comparison with 2020 levels. Although there exist nonbinding tools to provide information to developers that facilitate biodiversity‐friendly renewable developments (e.g., *Environmental Zoning of Renewable Energies: Wind and Photovoltaic* [MITECO, 2020]), these are focused on the network of important bird areas, the Natura 2000 network, and other areas of environmental significance, thus neglecting low‐productivity agricultural lands and grasslands, which usually host steppe bird populations (Medrano‐Vizcaíno et al., 2025). Therefore, a novel and refined prioritization tool that informs PV siting is key to reducing impacts on threatened steppe bird communities.

To gain a deeper understanding of the ecological processes that shape biodiversity and to refine conservation priorities, biodiversity should not be understood solely in terms of taxonomic diversity (TD) (Pollock et al., 2017). A thorough assessment must include other facets, such as evolutionary history (phylogenetic diversity [PD]) and functional diversity (FD). PD provides insights into the evolutionary uniqueness of species within a community (Lum et al., 2022), whereas FD focuses on the variety of functional traits in communities, which can be useful in assessing ecosystems’ resilience, stability, and functioning (Sol et al., 2020). An integrated analysis of TD, PD, and FD can help identify conservation‐valuable areas and potential conflicts with human activities from a more complete picture of biodiversity (Smiley et al., 2020). This approach can help pinpoint areas at high risk for biodiversity loss and guide decisions about where stricter protections are needed, being especially important for highly threatened communities.

We analyzed steppe birds as a case study in the context of the current worldwide expansion of PV energy by addressing two key questions: does a multifaceted prioritization approach (TD + FD + PD) outperform single‐facet strategies in retaining high biodiversity areas and areas with species of conservation concern and to what extent do PV infrastructures spatially overlap with high multifaceted steppe bird diversity (TD + FD + PD) in Spain? For this, we used the most recent data on the occurrence of 26 steppe bird species in Spain (III Spanish Atlas of Breeding Birds) (Medrano‐Vizcaíno et al., 2025; Molina et al., 2022) to identify high‐multifaceted‐diversity areas based on multiple biodiversity facets (TD, FD, and PD), thus ensuring the protection of individual species, as well as evolutionary distinctiveness and the diversity of the functional traits that support important ecosystem functions. We quantified the coverage of each facet that is retained when prioritized individually compared to when they are combined, thereby highlighting potential trade‐offs and complementarities among biodiversity dimensions. We also evaluated the effectiveness of each prioritization scenario (single‐facet and combined) in retaining areas with steppe birds of conservation concern. Finally, we overlapped the best prioritization scenario with a newly generated, spatially explicit map of installed PV infrastructures across Spain to identify exposure areas, where high multifaceted diversity areas coincide with high PV occupancy, and PV avoidance areas, that is, high multifaceted diversity areas with low or null PV occupancy, where future PV implantation should be avoided or minimized. Our results provide a framework that derives generalizable conclusions and recommendations and may be transferred to other taxa and regions.

## METHODS

### Study area and species occurrence

We performed our analyses across Peninsular Spain and the Balearic Islands, a territory that contains a great proportion of European steppe birds (Burfield et al., 2023). In particular, Iberia harbors steppe bird species distributed over different regions in the Palearctic, like the Mediterranean basin, Atlantic Europe, or central Asia, which confers high evolutionary and biogeographical singularity to the Iberian steppe avifauna (Santos & Suárez, 2005). As spatial units, we used Universal Transverse Mercator (UTM) grid cells of 10 × 10 km, which totaled 3838 cells that contained at least five species, which we considered the minimum number for meaningful FD analyses (see below).

We used occurrence data for 26 species (Appendix S1) that were included in previous steppe bird conservation research in Spain (Medrano‐Vizcaíno et al., 2025; Traba et al., 2007). These species are typical of Iberian natural steppes and agricultural pseudo‐steppes (Suárez et al., 1997; Traba et al., 2007), are essentially ground‐nesting, occur in treeless and mainly flat areas, and have their main European populations in the Iberian Peninsula (Medrano‐Vizcaíno et al., 2025). As for steppe habitats, we referred to natural and seminatural open habitats, including natural steppes, dry cereal farmlands, grasslands, and shrublands (Ollero & van Staalduinen, 2012). Data were compiled from the *III Spanish Atlas of Breeding Birds* (period 2014–2018) (Molina et al., 2022) and eBird (records during the breeding period, February–August 2019–2023); therefore, data covered a decade (details in Medrano‐Vizcaíno et al. [2025]).

### PV energy data

We gathered all available geospatial PV databases at global, national, and regional levels, which consisted of polygons or points, or both. We completed this information with the SatlasPreTrain geospatial data (Bastani et al., 2023), which consisted of a high‐accuracy AI‐generated output of polygons classified as solar energy facilities at a global scale. Additionally, we identified areas where spatially explicit data on PVs were missing based on the Spanish PRETOR database (MITECO, 2024b), which contained information on the installed PV power per municipality. Missing PVs were georeferenced with the aid of high‐resolution PNOA (Plan Nacional de Ortofotografía Aérea) images (Instituto Geográfico Nacional, 2024). For these georeferenced facilities, we generated points separated by approximately 250 m across each PV facility. Then, we applied a 250‐m buffer around each point (∼0.20 km^2^ per buffer), which was smaller than the mean PV area estimated per grid cell (∼0.49 km^2^). Overlapping buffers were subsequently dissolved to generate a single polygon representing each PV facility. Although this procedure may have underestimated true PV areas, as circular buffers do not fully capture the rectilinear geometry typical of PV installations, it avoided overestimating the extent of PV infrastructure.

### Biodiversity facets analysis

Taxonomic diversity was calculated as the number of species present in each cell. FD was calculated using the FRic (functional richness) index, which quantifies the amount of functional space occupied by the species of a given community (Villéger et al., 2008). This index is more accurate than the dendrogram‐based index and is comparable with species richness and the Faith's PD index for PD, as all three reflect the extent of diversity based on richness, whether taxonomic, functional, or phylogenetic (Maire et al., 2015; Tucker et al., 2017). To this end, for each species, we gathered data on 32 traits related to morphometry, body mass, life history, habitat, diet, population density, and migratory behavior from different sources, such as databases (e.g., Cooke et al., 2019; Santini et al., 2023; Tobias et al., 2022), books (e.g., Madroño et al., 2004; Snow & Perrins, 1998), scientific articles (e.g., Pérez‐Granados et al., 2013; Tsuboi et al., 2018), and our own field data (complete list of species and traits in Appendix S1). We encountered missing data for some traits: birth or hatching weight (10 missing values), brain size (eight missing values), habitat breadth (two missing values), degree of development (one missing value), and population density (three missing values). To avoid removing traits or species with incomplete data, we imputed missing values, relying on the fact that traits are usually correlated (Johnson et al., 2021). Imputation was performed in R (R Core Team, 2022) with the function missforest from the package missforest, an approach that performs better than other methods, such as *k*‐nearest neighbor imputation or multivariate imputation (Stekhoven, 2013; Tucker et al., 2017).

We built a species × trait matrix (i.e., 26 rows × 32 columns), and a community × species matrix (i.e., 3838 rows × 26 columns). This latter matrix represented the presence/absence of each species in each grid cell. We used a minimum threshold of five species per cell because the number of species in each one must be higher than the number of dimensions used to define the multidimensional functional space based on principal coordinates analysis axes (we selected four dimensions, which accounted for 88% of the total variance based on the relative eigenvalues [Appendix S2]). As including additional dimensions would have resulted in a significant loss of data (more grid cells excluded from analyses), the selection of four dimensions ensured a meaningful representation of the functional space while retaining as many cells as possible, thus minimizing data loss.

Given that the FRic index is highly influenced by TD (i.e., a higher number of species can lead to a broader range of traits, depending on species trait variation) (Plass‐Johnson et al., 2016), we calculated the standardized effect size of FRic (SESFRic) to remove the effect of species richness. The SESFRic was calculated as (observed FRic − mean of expected FRic) / (SD of expected FRic), where observed FRic is the observed values of FRic, and the mean and SD of expected FRic are values obtained from null models (Mason et al., 2013). To this end, we executed 1000 permutations of the community matrix using the independent swap null model, which randomized the data matrix and maintained the original species richness (Aros‐Mualín et al., 2021). We used the function dbFD from the R package FD (Laliberté et al., 2014) to calculate FRic and the function randomizeMatrix from the R package *picante* (Kembel et al., 2010) to generate the null models.

To calculate PD, we used the bird phylogeny of Jetz et al. (2012) and applied the Faith's PD index, which represented the sum of phylogenetic branch lengths connecting species within a community (Faith, 1992). As for FRic, this index was highly sensitive to TD; therefore, we used the function ses.pd from the R package picante to calculate the effect size of PD (SESPD) based on 1000 permutations that randomized species labels while preserving the underlying phylogenetic structure. The use of standardized effect sizes minimized correlations among biodiversity facets (Appendix S3).

To compare analyses, all three biodiversity facet values—TD, SESFD (hereafter FD), and SESPD (hereafter PD)—were scaled between 0 and 1 by subtracting the minimum and dividing by the difference between the maximum and the minimum. As an additional analysis, we assessed the contribution of each species to the overall FD and PD. For this, we iteratively removed each species and recalculated the resulting FD and PD. The contribution of each species was computed as the difference between the total FD and PD with all species and the FD and PD obtained without that species.

### Spatial prioritization scenarios

We used the spatial prioritization software Zonation 5 (Moilanen et al., 2022) to generate four prioritization scenarios: three representing single biodiversity facets (TD, FD, and PD) and one combining all three facets (TD + FD + PD), where TD, FD, and PD were assigned equal importance by assigning an equal weight of 1. Zonation 5 produced a hierarchical prioritization of sites (cells) based on their biological value, accounting for complementarity by considering the representation level of biodiversity features (i.e., biodiversity facets in our study) (Moilanen et al., 2022).

We selected the core‐area zonation 2 (CAZ2) marginal loss rule, which enhances coverage for underrepresented features at the cost of a slight reduction in overall average coverage (Moilanen et al., 2022). This rule performs better than other marginal loss rules, such as ABF (additive‐benefit function) and CAZMAX (core‐area zonation); ABF prioritizes higher average coverage at the expense of underrepresented features, whereas CAZMAX results in lower overall average coverage (Moilanen et al., 2022).

To assess the effectiveness of each scenario in preserving TD, FD, and PD, we overlaid the top 30% of TD, FD, and PD cells (separately) over the top 30% of each of the four prioritization scenarios (i.e., TD, FD, PD, and TD + FD + PD) and calculated the retention of the three biodiversity facets under each prioritization scenario. This was useful to quantify to what extent TD, FD, and PD are preserved under each scenario.

We also analyzed the coverage of these scenarios over areas inhabited by steppe bird species of conservation concern (threatened species areas, hereafter). To this end, we used the occurrence data of the 26 steppe bird species to derive a threatened species index map based on the combination of three threat status categories at European and Spanish levels: the European population status (EPS) (BirdLife International, 2004; Burfield et al., 2023), the species of European conservation concern (SPEC) (BirdLife International, 2004; Burfield et al., 2023), and the Spanish Catalogue of Threatened Species category, which was the official and legal instrument for the protection of wild species in Spain (MITECO, 2023). Following Medrano‐Vizcaíno et al. (2025), we applied a numeric scoring scale that ranged from 0 to 10, where 10 represented the highest concern category (Appendix S4). The individual threat status scores for each of the three sources were summed across species per grid cell and standardized from 0 to 1. Finally, the standardized scores were summed to generate the threatened species index per cell.

Next, we overlaid the top 30% of each prioritization scenario over the top 30% of the threatened species index (i.e., threatened species areas) (Appendix S5) to calculate the coverage of each prioritization scenario over regions hosting a high number of threatened species. The best prioritization scenario was selected based on the highest average retention and more balanced values across facets. Additionally, we conducted pairwise combinations of diversity facets (i.e., TD + FD, TD + PD, and FD + PD) to facilitate comparison among all combinations of biodiversity facets (Appendix S6).

We focused on the 10 × 10‐km grid cells with the top 30% values of each biodiversity facet, aligning with the targets set by the Kunming–Montreal Global Biodiversity Framework (2022) and the EU Biodiversity Strategy (European Commission, 2020), both of which advocated conserving at least 30% of global and European land areas by 2030. As all grid cells represented equal areas, the top 30% of values corresponded approximately to 30% of the study area, providing a spatially consistent way to identify priority areas for biodiversity conservation.

### Steppe birds’ exposure to PV facilities

To assess the current and potential exposure of steppe birds to PV infrastructures, we overlaid the multifaceted diversity map generated by Zonation 5 (i.e., the TD + FD + PD scenario) with PV occupancy (the proportion of land within each grid cell currently covered by PV infrastructure). Both multifaceted diversity and PV occupancy values were divided into tertiles (low, medium, high). The PV occupancy was standardized to a 0–1 scale (min–max scaling) to align with the range of the multifaceted diversity map. Original PV occupancy ranged from <0.01% to 13.14%. Tertile boundaries were determined based on the distribution of observed values, with high occupancy defined as 2.78–13.14%, medium as 0.32–2.77%, and low as <0.01–0.32%.

The combination of the PV occupancy and multifaceted diversity maps, based on their tertile values, resulted in nine joint categories (i.e., high multifaceted diversity, high PV occupancy, high multifaceted diversity, medium PV occupancy, etc.) that were represented in bivariate maps produced with the R biscale package (Prener et al., 2022). We identified as exposure areas those cells with high values (upper tertile) of both multifaceted diversity and PV occupancy maps. On the other hand, cells with high multifaceted diversity values and low current PV occupancy were identified as PV avoidance areas: these represent locations where future developments should be avoided or minimized in order to avoid potential impacts on steppe birds.

We presented our results both at the national level and at the level of administrative regions because, in Spain, the latter have the legal competences on environmental policy and PV approval when plants have an installed capacity of <50 MW (IEA, 2021).

## RESULTS

**FIGURE 1:**
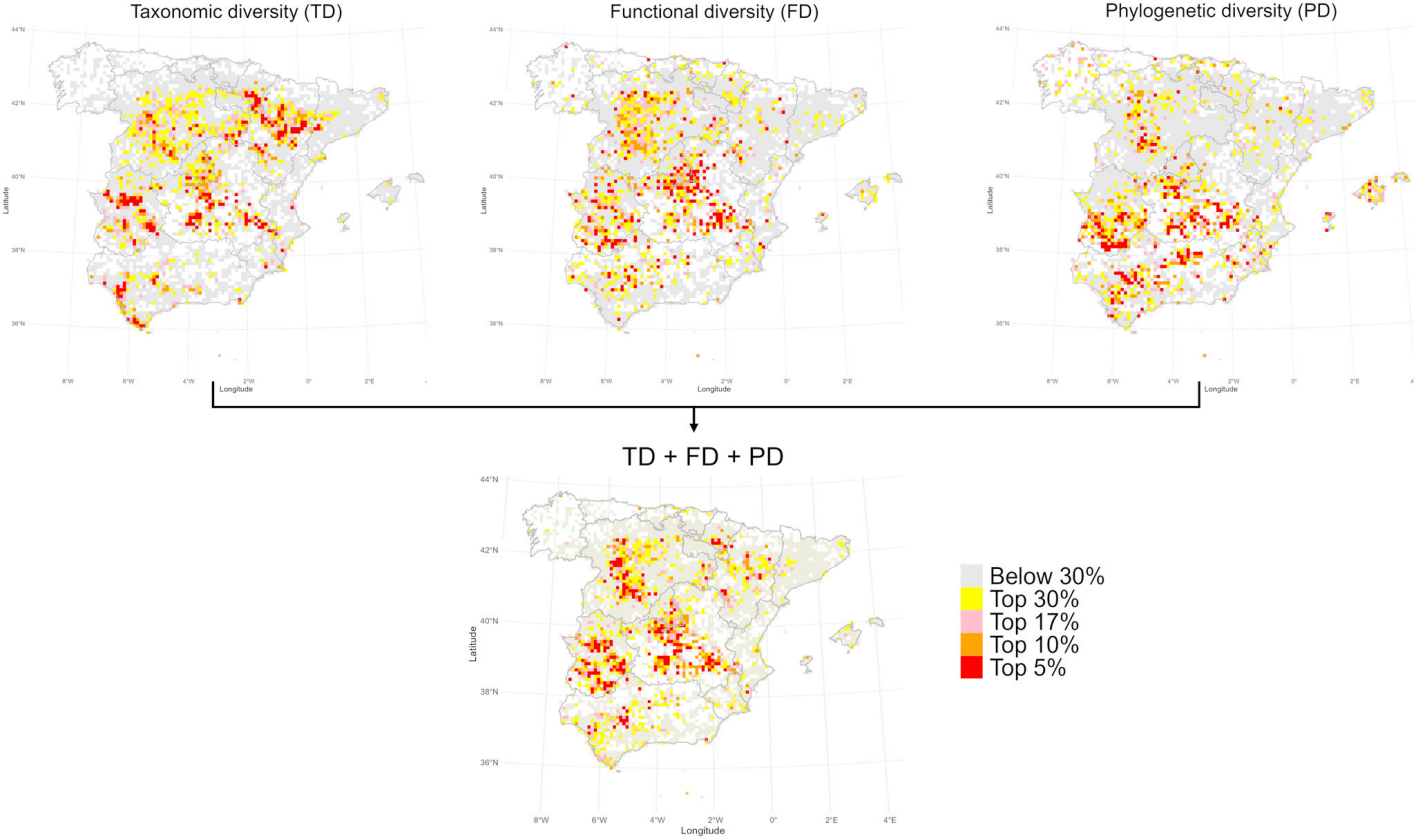
Areas of conservation priority in Spain and Balearic Islands based on steppe bird taxonomic (TD), functional (FD), and phylogenetic (PD) diversity assessed separately and in combination. Percentages indicate the proportion of land corresponding to the highest priority areas (30% [Kunming–Montreal Global Biodiversity Framework, 2022; European Commission, 2020], 17% [CBD, 2010], 10%, and 5%).

We found marked differences in spatial patterns among biodiversity facets (Figure 1). Notably, a high diversity of any facet did not imply high diversity for other facets, with Pearson correlation coefficients ranging between −0.1 and 0.5 (Appendix S3). For example, northern Spain and some regions in western Spain showed a high TD only, whereas regions in southern Spain showed particular areas with an increased PD only. Likewise, some regions in central Spain showed areas with high FD only. Also, insular areas appeared more important under the PD scenario. The contribution of individual species to both FD and PD varied considerably (Appendices S7 & S8). The great bustard (*Otis tarda*), the zitting cisticola (*Cisticola juncidis*), and the Eurasian stone‐curlew (*Burhinus oedicnemus*) showed the highest contributions to FD (i.e., their removal resulted in the largest FD reduction). Regarding PD, the species with the highest contributions were the short‐eared owl (*Asio flammeus*), the Eurasian stone‐curlew, and the lesser kestrel (*Falco naumanni*).

According to Zonation 5, the most inclusive conservation scenario was TD + FD + PD, as it covered areas that remained unprioritized in the other scenarios. This scenario included areas in central and western Spain that were not highly prioritized under the PD scenario; areas in northeastern Spain that were not prioritized under the FD and PD scenarios; and some areas in central Spain that were not prioritized under the TD and PD scenarios. Interestingly, regions along the northern and northwestern borders of the country were not prioritized under any scenario (Figure 1).

**FIGURE 2:**
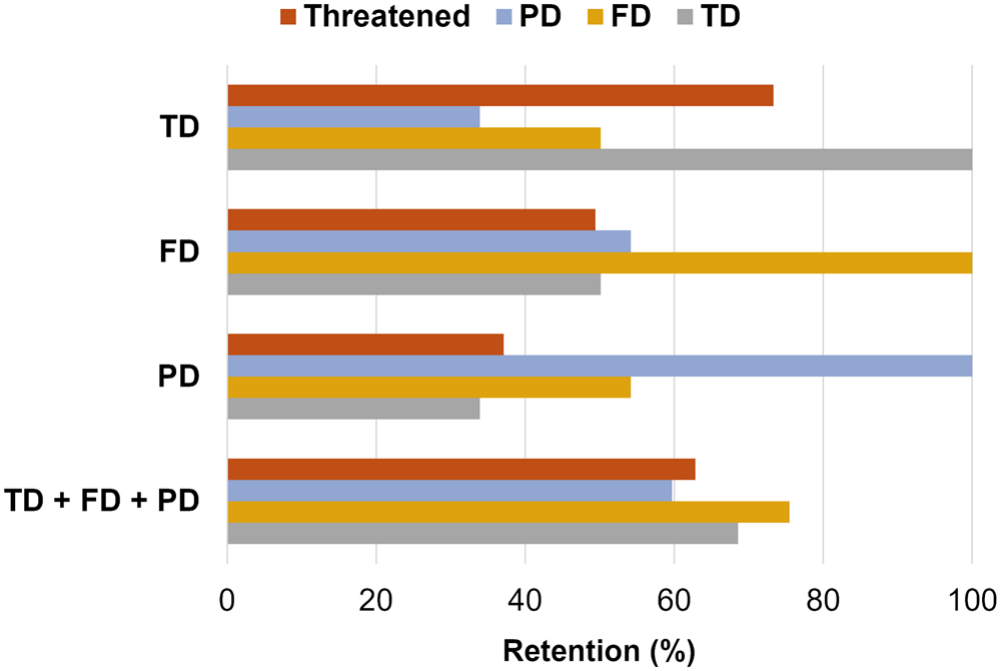
Percentage of taxonomic diversity (TD), functional diversity (FD), phylogenetic diversity (PD), and threatened species index retained under the four steppe bird conservation prioritization scenarios (*y*‐axis).

We found that the TD + FD + PD scenario retained the highest overall proportion of biodiversity facets and areas inhabited by threatened species (Figure 2). This scenario achieved an overall retention of 66.6%, including 68.5% of TD, 75.5% of FD, 59.7% of PD, and 62.8% of threatened species areas. It was followed by the TD scenario, with an overall retention of 64.3%, although inflated by its evident 100% coverage of TD, and showing poor representation of FD (50.1%) and PD (33.9%), despite high coverage of areas with threatened species (73.3%). The FD scenario performed worse, retaining 50.1% of TD, 54.2% of PD, and 49.4% of threatened species areas. The PD scenario showed even lower and more unbalanced retention percentages, with 33.9% of TD, 54.2% of FD, and 37.1% of threatened species areas. Overall, the TD + FD + PD scenario achieved the highest average retention and more balanced values across facets when compared with the two‐facet scenarios (Appendix S6).

### Steppe birds’ current exposure to solar PV facilities, and PV avoidance areas

**FIGURE 3:**
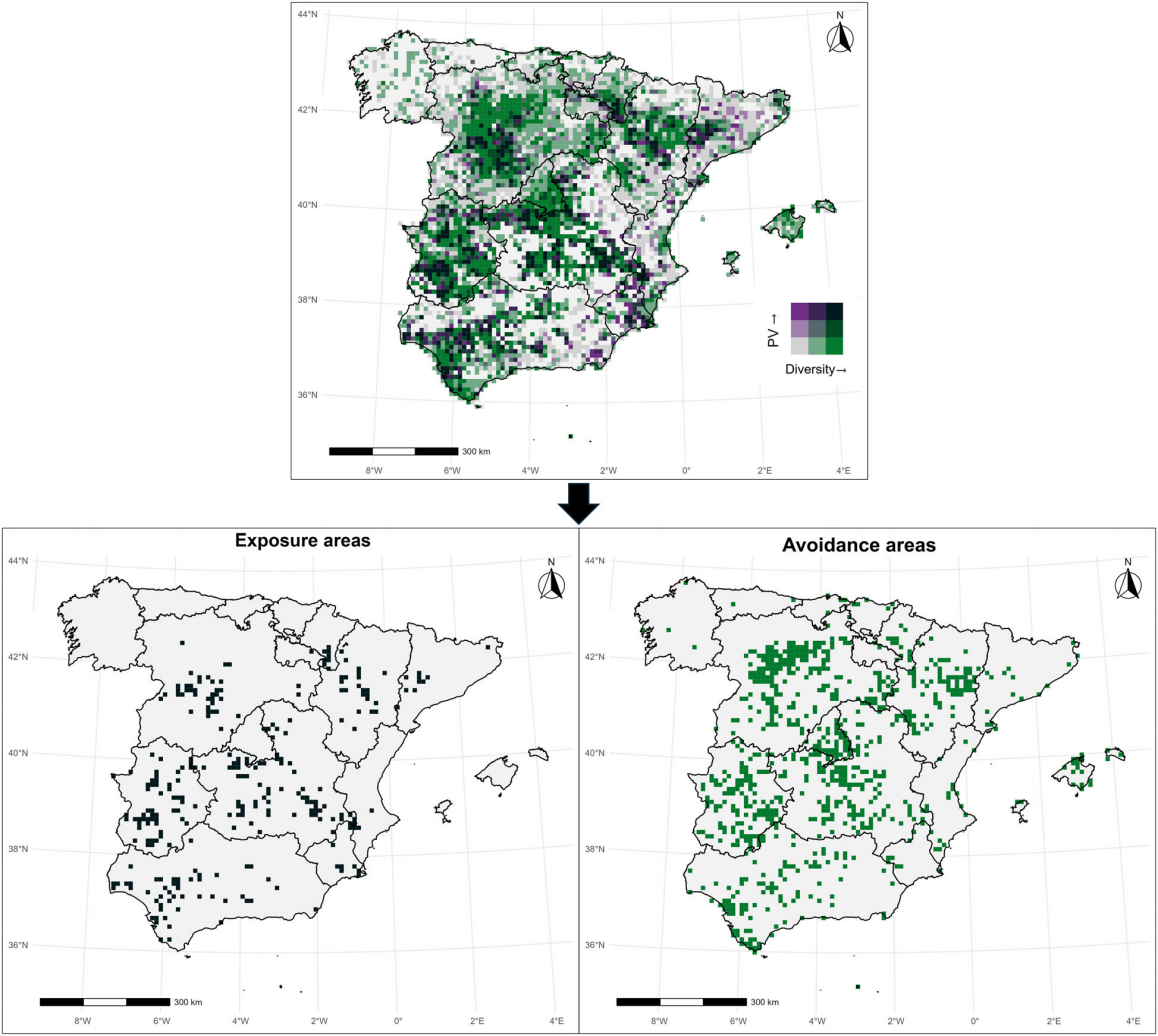
Identification of priority areas for the conservation of steppe birds in peninsular Spain and Balearic Islands: (top) bivariate map combining low, medium, and high values of multifaceted diversity (combination of taxonomic, functional, and phylogenetic diversity) and photovoltaic infrastructure (PV) occupancy and (bottom) exposure and PV avoidance areas extracted from the bivariate map.

We found that 40.5% of all cells and 53.1% of high multifaceted diversity cells host PV facilities. The intersection between the multifaceted diversity and PV occupancy maps revealed distinct patterns of exposure of steppe bird diversity to solar energy development (Figure 3). We detected 276 exposure cells having both high multifaceted diversity and high PV occupancy (7.2% of our study area). Exposure cells were mainly concentrated in central, southern, and northwestern Spain. Here, steppe bird communities are already under pressure from existing PV development, highlighting potential biodiversity–energy conflicts that require conservation attention.

In addition, we identified 765 cells having high multifaceted diversity but low current PV occupancy (19.9% of our study area), being broadly distributed across Spain; however, they showed higher concentration in northern, central, and central‐western Spain. These PV avoidance areas represent locations where future infrastructures should be avoided or minimized, as they could pose risks to steppe bird communities if not carefully managed.

## DISCUSSION

The rapid global expansion of renewable energy infrastructure is transforming landscapes at an unprecedented pace, often at the expense of biodiversity. The conversion of natural habitats for renewable energy projects poses a critical threat to ecosystems worldwide, exacerbating the ongoing biodiversity crisis (IPBES, 2019). Reconciling biodiversity conservation and climate change mitigation goals is a societal and political challenge. We addressed this global issue by examining spatial conflicts between PV development and steppe bird conservation in Spain, a key stronghold for these species. Our multidimensional analysis, integrating TD, FD, and PD, revealed numerous regions where high biodiversity value coincided with substantial PV infrastructure, highlighting areas at risk from current and future PV developments.

These findings underscore that relying on a single biodiversity facet can overlook critical conservation priorities. By adopting a multidimensional approach, our study provides a robust framework for identifying potential conflicts between renewable energy projects and biodiversity conservation, offering a scalable strategy applicable to threatened ecosystems worldwide. Additionally, the proposed approach can be extended to other infrastructures (e.g., wind farms) and species groups.

### Spatial patterns of steppe bird biodiversity facets

Previous studies have emphasized the role of ecological and evolutionary processes in shaping biodiversity patterns across geographic regions (Monnet et al., 2014). This aligns with our results showing that, although central and western regions in Iberia are taxonomically, functionally, and phylogenetically highly diverse in terms of steppe birds, other regions, such as parts of the northeastern interior, emerged as important mainly for taxonomic diversity. In contrast, several areas in southern Spain stand out primarily for their high PD. These patterns suggest that some areas, although rich in species, support communities composed of species that are functionally similar and evolutionarily close. This is indicative of low ecological differentiation and limited evolutionary distinctiveness, which may reduce the resilience of communities to environmental change (Cadotte et al., 2012).

For example, areas with high TD but low FD and PD might be characterized by a prevalence of species‐rich taxonomic groups (e.g., certain families of passerines) that, although numerous, share similar functional traits and a relatively recent common evolutionary history. This can result in higher functional redundancy (where many species perform similar ecological roles) and shorter overall phylogenetic branch lengths, meaning the loss of any single species in such a community might result in a comparatively small reduction in functional volume or phylogenetic distinctiveness. On the other hand, areas concentrating high values of all three biodiversity facets represent critical conservation priorities, as they host not only many species but also deeper evolutionary lineages and a broader range of ecological strategies, from large ground‐dwelling herbivores (great and little bustards) to small‐sized invertivores (Dupont's lark or wheatears) and medium‐sized raptors (harriers).

Recognizing these multidimensional patterns is essential for anticipating how different human pressures may affect biodiversity in various ways. For example, intensive agriculture has been shown to reduce FD in farmland birds (Guerrero et al., 2024), a concern for steppe birds that largely rely on cereal steppes. Hunting pressure, habitat destruction, and land‐use changes have been associated with declines in TD, FD, and PD, respectively (Romero‐Muñoz et al., 2021). Similarly, climate change is expected to negatively affect FD (Penjor et al., 2022), whereas urbanization poses risks particularly to PD (La Sorte et al., 2018). Recently, Gómez‐Catasús et al. (2025) identified that threatened European steppe birds are mostly large‐sized, long‐lived, ground‐dwelling, and ground‐foraging species, providing insights into how agriculture intensification and human‐induced mortality are eroding nonrandom sections of their functional space.

### Spatial prioritization

Protecting multiple facets of biodiversity presents significant challenges, as it often involves unavoidable trade‐offs (Wilson & Law, 2016). No prioritization scenario was able to fully preserve all three biodiversity facets. However, our results show that integrating TD, FD, and PD maximizes the overall coverage of biodiversity facets and areas with high numbers of threatened species. This scenario (TD + FD + PD) retained almost 60% of PD, 75% of FD, nearly 70% of TD, and more than 60% of regions with high numbers of threatened species, capturing the complex interactions between threatened species, their ecological roles, and their evolutionary history, key factors for conservation planning (Brum et al., 2017; Pollock et al., 2017).

Our results demonstrated that prioritization scenarios based on a single biodiversity facet were not effective at preserving other facets. However, traditional conservation strategies and legislation have focused predominantly on TD, neglecting functionally and evolutionarily diverse regions (Cadotte & Tucker, 2018). For example, the TD scenario showed the worst performance among all scenarios, covering only one third of PD and half of FD. This highlights a critical issue: a TD‐centric approach can overlook key areas that, although perhaps not maximizing species numbers, are crucial for other biodiversity dimensions. For instance, such an approach might undervalue regions where the presence of species with unique functional traits (e.g., the great bustard, zitting cisticola, and Eurasian stone‐curlew) is vital for maintaining functionally diverse ecosystems. Similarly, areas harboring species that represent particularly distinct evolutionary lineages (e.g., the short‐eared owl, Eurasian stone‐curlew, and lesser kestrel, as evidenced in the community's phylogenetic tree) (Appendix S9) could be missed, leading to a significant loss of PD. These examples illustrate a broader principle applicable to many ecosystems: conserving the full spectrum of biodiversity requires looking beyond simple species counts.

Conservation programs often require balancing competing demands, such as habitat restoration and the mitigation of human impacts, while adhering to financial constraints and insufficient political will. Therefore, a key consideration for conservationists is how to effectively allocate limited resources to achieve the greatest possible benefits. Our prioritization approach, while emphasizing areas with high biodiversity across the three facets, also aimed to improve coverage of underrepresented biodiversity facets across grid cells by using the CAZ2 marginal loss rule. This differs from more restrictive prioritization approaches in Zonation 5 software (e.g., ABF and CAZMAX), which have a lower distribution coverage of underrepresented features (in this case, biodiversity facets) (Moilanen et al., 2022). These latter prioritization approaches would have, for instance, excluded areas with high FD and PD but low TD. Conserving these areas, where species richness may be low but harbor species with unique evolutionary lineages that play key ecological roles, is crucial, as their loss could cause disproportionate ecosystem disruptions (Rosauer et al., 2009). These findings highlight the necessity of a multidimensional conservation approach to ensure the long‐term sustainability of species assemblages and ecosystem functions. Although identifying priority areas is a crucial step, translating this knowledge into effective spatial planning is equally important, especially in the context of rapid PV expansion.

### Conflicts between steppe bird conservation and PV infrastructures

Strategic planning is urgently needed to balance renewable energy expansion and biodiversity conservation. In Spain, the rapid growth of PV infrastructures, whose sites are initially selected based on the adequate conditions for the developers (i.e., good connections with electric grid distribution, good conditions for producing solar energy, and landowners willing to negotiate with the developers), is increasingly threatening steppe bird habitats, posing a major risk to already declining steppe bird populations (MITECO, 2024a; Serrano et al., 2020). In that sense, the large proportion of exposure areas (hosting both rich steppe bird biodiversity areas and PV infrastructures) mostly points to conditions for both steppe birds and PV infrastructures being coincident, rather than to coexistence of birds with PV farms.

Our analysis provided a clear framework for identifying areas where PV developments could threaten steppe bird biodiversity, allowing for informed decision‐making. The representation of different levels of multifaceted diversity and PV occupancy to identify exposure and PV avoidance areas is important because the effects of PV developments are not uniform across all areas. For example, although the area occupied by PV plants in some regions may seem small, the localized effects on biodiversity can be severe. Unless conservation planning is effectively implemented, there is a high risk that high‐diversity areas will be exposed to meet the 76,000 MW target by 2030 (57,000 MW of utility‐scale PVs and 19,000 MW of self‐consumption; MITECO, 2024a).

Steppe birds are already under considerable pressure due to agricultural intensification, abandonment of extensive grazing, and human‐induced mortality due to agricultural practices and collisions with powerlines, factors that have led their populations to decline worldwide (Gómez‐Catasús et al., 2025; Traba & Morales, 2019; Traba & Pérez‐Granados, 2022). An uninformed and disproportional PV infrastructure expansion can be an additive highly threatening factor, especially in central, central‐western, northern interior, and southern Spain, where we identified most current exposure and PV avoidance cells. Indeed, some regions in southern and central‐western Spain have the highest installed capacity and generated PV power in Spain (Red Eléctrica de España, 2025). Without targeted conservation efforts, large‐scale PV developments in these areas could result in substantial habitat loss, increased mortality due to collisions with PV panels and distribution lines, and disruptions to ecosystem functions (Dhar et al., 2020; Lambert et al., 2022; Moscatelli et al., 2022).

We consider that periodic conservation assessments and targeted habitat restoration initiatives, particularly in identified exposure areas (where PV infrastructure is already installed, and where potential impacts on birds may be ongoing), could be beneficial for wildlife. Concurrently, policies limiting PV developments in both exposure and designated PV avoidance areas are crucial for safeguarding steppe bird populations. However, achieving a balance between energy development and biodiversity conservation involves careful planning in potentially conflicting areas and identifying and incentivizing PV development in locations with lower ecological value. An ideal approach could integrate our prioritization findings with ecovoltaic facilities and the strategic siting of PV plants on degraded lands (e.g., former quarries, mines, landfills) or within the urban areas (e.g., rooftops of industrial or commercial buildings), thereby minimizing new incursions into sensitive habitats. Ecovoltaic facilities are designed based on ecologically informed strategies that balance energy generation with the preservation and enhancement of ecosystem services (Sturchio & Knapp, 2023), focusing on principles like site selection, park layout, ecosystem design, and adaptive ecosystem management (Tölgyesi et al., 2023). Research on this topic is scarce, with one previous study reporting higher richness of grassland birds in ecovoltaic sites in comparison with areas without ecovoltaics (Walston et al., 2025). Our results offer valuable insights to avoid the establishment of PVs in areas of high conservation value and instead establish ecovoltaics that can offer potential benefits for conservation, contingent on evaluation of their effectiveness via monitoring programs during construction and operation.

### Limitations and future research

Despite the comprehensive nature of our study, there are several limitations that warrant attention. First, the responses of species to PV infrastructure can vary depending on local ecological conditions, species behavior, and the magnitude of infrastructure. There is a major knowledge gap in understanding how different species respond to the installation of PV plants in terms of direct impacts (e.g., habitat loss and collisions) and indirect effects (e.g., changes in food availability and provision of breeding habitats). Understanding the interplay of PV plants with variables such as space use, reproductive success, physiological levels, or type of flight can provide additional and relevant clues for conservation.

Another important aspect is the evaluation of the potential impacts of emerging energy sources and associated infrastructures (Vermeylen, 2010). Such efforts are essential to understand the cumulative effects of renewable energy development on biodiversity. Importantly, these impacts may not occur in isolation but rather interact synergistically with other well‐documented threats to steppe birds, such as agricultural intensification, habitat loss, power line collisions, hunting, and climate change (Gómez‐Catasús et al., 2025; Serrano et al., 2020). Assessing these combined pressures is critical for designing effective, evidence‐based conservation strategies that can mitigate the compounding effects on already vulnerable species and ecosystems.

To our knowledge, our database of PV infrastructure is the most accurate to date in Spain. We are confident that it captures most PV plants >5 MW and many more with 1 MW installed power. Ideally, this information should be provided and updated regularly by regional governments, which is essential in the case of Spain because the regional governments hold the responsibility to approve PV developments, as well as the responsibility of preserving vulnerable species. In any case, the spatial analysis of our national‐scale PV database, together with data on a threatened group, such as steppe birds, highlights opportunities to minimize potential biodiversity impacts and for strategic energy planning.

Our approach could be directly integrated into Spain's policy and regulatory frameworks for PV development. Currently, PV sites are selected based on factors favorable to developers (e.g., grid connections, solar potential, and land availability). As environmental impact assessments are mandatory (BOE, 2023), our prioritization maps can guide regional authorities during early site evaluation. This tool complements and refines MITECO's environmental zoning system, aligning with the impact mitigation hierarchy (Phalan et al., 2018). At broader scales, it can inform regional and national energy policies, such as REPowerEU, the Fit for 55 package (European Commission, 2022), and the PNIEC 2023–2030 (MITECO, 2024a), which is the national strategic plan that sets out the roadmap for climate and energy policy until 2030. More immediately, our maps provide a valuable resource for designating Renewable Acceleration Areas, required by early 2026 (European Commission, 2022). Nevertheless, the spatial scale used and the quality and phenology of the data (Atlas and eBird data, which do not arise from standardized sampling in the whole territory and may include information gaps) may be too coarse to ensure truly efficient spatial planning for PV avoidance areas at small spatial extents, which would still require detailed, local‐scale environmental impact assessments.

The rapid expansion of renewable energy facilities is essential to achieve global climate change mitigation goals. However, this expansion needs careful management to avoid exacerbating the loss of biodiversity, particularly in regions that support threatened species. In many countries, such as Spain, PV infrastructure is being developed in highly suitable areas for steppe birds. Our analyses exemplify the challenges faced by wildlife adapted to open, flat, low‐productivity areas under a scenario of swift solar PV development, and how spatial prioritization may help reconcile climate change mitigation and biodiversity conservation goals. Our findings provide valuable insights for policymakers, energy developers, and conservationists, offering a framework for balancing renewable energy expansion with biodiversity conservation.

Our framework is applicable to other regions and species. For example, the use of comprehensive global trait databases (necessary for FD analyses) available for different taxa, such as birds (Tobias et al., 2022), mammals (Soria et al., 2021), amphibians (Oliveira et al., 2017), and reptiles (Oskyrko et al., 2024), can facilitate assessments of the impact of other infrastructures, such as roads (Rahhal et al., 2023) and wind energy facilities (Thaxter et al., 2017). This is crucial in biodiversity hotspots, where thousands of species may be affected by human development (Medrano‐Vizcaíno et al., 2022), yet distribution and trait data can be incomplete or incorrectly assumed for some species (e.g., Medrano‐Vizcaíno & Rueda, 2018).

By using an approach based on TD, FD, and PD to assess potential conflicts with human infrastructure, we can better understand the ecological consequences of human‐induced environmental changes and develop strategies that promote both sustainability and biodiversity conservation.

## Supporting information



Supplementary Material

Supplementary Material

## Data Availability

Trait data are in Appendix S1, and R code and spatial data are available from https://github.com/pabmevi/BiodiversityFacets_ConsBio. Occurrence data for each species can be made available upon request to the authors and SEO BirdLife.
